# High-pressure balloon assessment of pelviureteric junction prior to laparoscopic “vascular hitch”

**DOI:** 10.1590/S1677-5538.IBJU.2015.0343

**Published:** 2016

**Authors:** Alberto Parente, José-María Angulo, Rosa Romero, Laura Burgos, Rubén Ortiz

**Affiliations:** 1 Departamento de Urología Pediátrica, Hospital Universitario Gregorio Marañón, Madrid, España

**Keywords:** Multicystic renal dysplasia, bilateral [Supplementary Concept], Laparoscopy, Child

## Abstract

**Aim:**

To assess if calibration of the ureteropelvic junction (UPJ) using a high-pressure balloon inflated at the UPJ level in patients with suspected crossing vessels (CV) could differentiate between intrinsic and extrinsic stenosis prior to laparoscopic vascular hitch (VH).

**Materials and Methods:**

We reviewed patients with UPJO diagnosed at childhood or adolescence without previous evidence of antenatal or infant hydronephrosis (10 patients). By cystoscopy, a high-pressure balloon is sited at the UPJ and the balloon inflated to 8-12 atm under radiological screening. We considered intrinsic PUJO to be present where a ‘waist’ was observed at the PUJ on inflation of the balloon and a laparoscopic dismembered pyeloplasty is performed When no ‘waist’ is observed we considered this to represent extrinsic stenosis and a laparoscopic VH was performed. Patients with absence of intrinsic PUJ stenosis documented with this method are included for the study.

**Results:**

Six patients presented pure extrinsic stenosis. The mean age at presentation was 10.8 years. Mean duration of surgery was 99 min and mean hospital stay was 24 hours in all cases. We found no intraoperative or postoperative complications. All children remain symptoms free at a mean follow up of 14 months. Ultrasound and renogram improved in all cases.

**Conclusion:**

When no ‘waist’ is observed we considered this to represent extrinsic stenosis and a laparoscopic VH was performed. In these patients, laparoscopic transposition of lower pole crossing vessels (‘vascular hitch’) may be a safe and reliable surgical technique.

## INTRODUCTION

The incidence of crossing vessels (CV) in the etiology of Ureteropelvic Junction Obstruction (UPJO) in children ranges from 11% to 15% (1), but has been reported as frequently as 58% in a series of older children with symptomatic UPJO and normal prenatal ultrasonography (2). This is especially common in children who report a history of intermittent abdominal pain coincident with abundant fluid ingestion and preserved renal function.

So far, the gold-standard treatment for these patients is pyeloplasty, with laparoscopic pyeloplasty (LP) being recommended in older children. Recently however, cranial relocation of the lower pole crossing vessels or “vascular-hitch” (VH) (3-6) as described by Hellström (3) has gained popularity. The main limitation of vascular hitch is the difficulty in distinguishing between intrinsic and extrinsic stenosis in order to select the least invasive surgical option.

Although laparoscopic pyeloplasty is a common, low morbidity technique, VH provides its own benefit, namely a reduction in operative time and preservation of the intact urinary tract that removes the risk of urinary leakage and anastomotic stricture formation. Furthermore, drains or stents are not required, reducing the length of stay and avoiding the need for a later cystoscopic stent removal.

We propose that calibration of the ureteropelvic junction (UPJ) be performed using a high-pressure balloon inflated at the UPJ level in patients with suspected CV to differentiate between intrinsic and extrinsic stenosis. Those patients with demonstrated extrinsic outflow obstruction may benefit from VH and the surgeon will be able to be more confident in the ability to select only the required intervention at the time of surgery.

## MATERIALS AND METHODS

We reviewed patients with UPJO diagnosed at childhood or adolescence without previous evidence of antenatal or infant hydronephrosis. Those with a diagnosis of crossing vessels made by magnetic resonance urography (MRU) were included. Diagnosis of UPJO was made by ultrasound and diuretic renogram. When the imaging and clinical assessment were consistent with the existence of crossing vessels, MRU was performed as per departmental protocol, namely children over 4 years with no antenatal or perinatal history of hydronephrosis, with episodes of lumbar pain, evidence of crossing vessels on ultrasound or fluctuating hydronephrosis.

Surgical intervention was considered in the presence of loin or lumbar pain with intermittent obstructive hydronephrosis and/or UTIs, with hydronephrosis grade III or IV on ultrasound and the presence of an obstructive renogram.

When the MRU demonstrated CV a decision was made to treat with laparoscopic dismembered pyeloplasty or laparoscopic vascular hitch based on the PUJ calibration (intrinsic stenosis versus extrinsic).

Patients presenting with severe loss of renal function or other associated anomalies were excluded.

### Calibration

Under cystoscopic guidance a retrograde pyelography is performed. A 0.014’’ureteral guidewire is then introduced into the renal pelvis. A high-pressure balloon (Rx Muso® Terumo Corp., Somerset, NJ, USA) is sited at the UPJ and the balloon inflated to 8-12atm under radiological screening.

**Figure 1:**
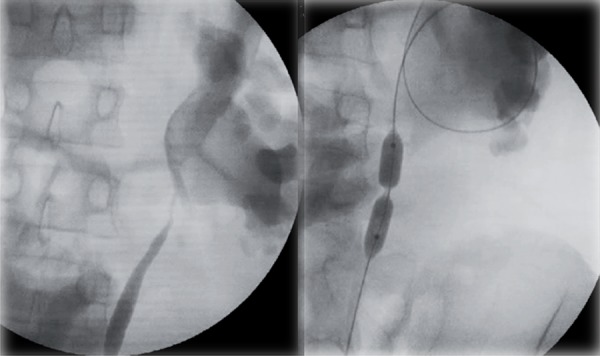
We considered intrinsic PUJO to be present when a ‘waist’ was observed at the PUJ on inflation of the balloon.

We considered intrinsic PUJO to be present when a ‘waist’ was observed at the PUJ on inflation of the balloon (Figure-1). A laparoscopic dismembered pyeloplasty was then performed as a continuation of this procedure under the same general anaesthetic.

**Figure 2:**
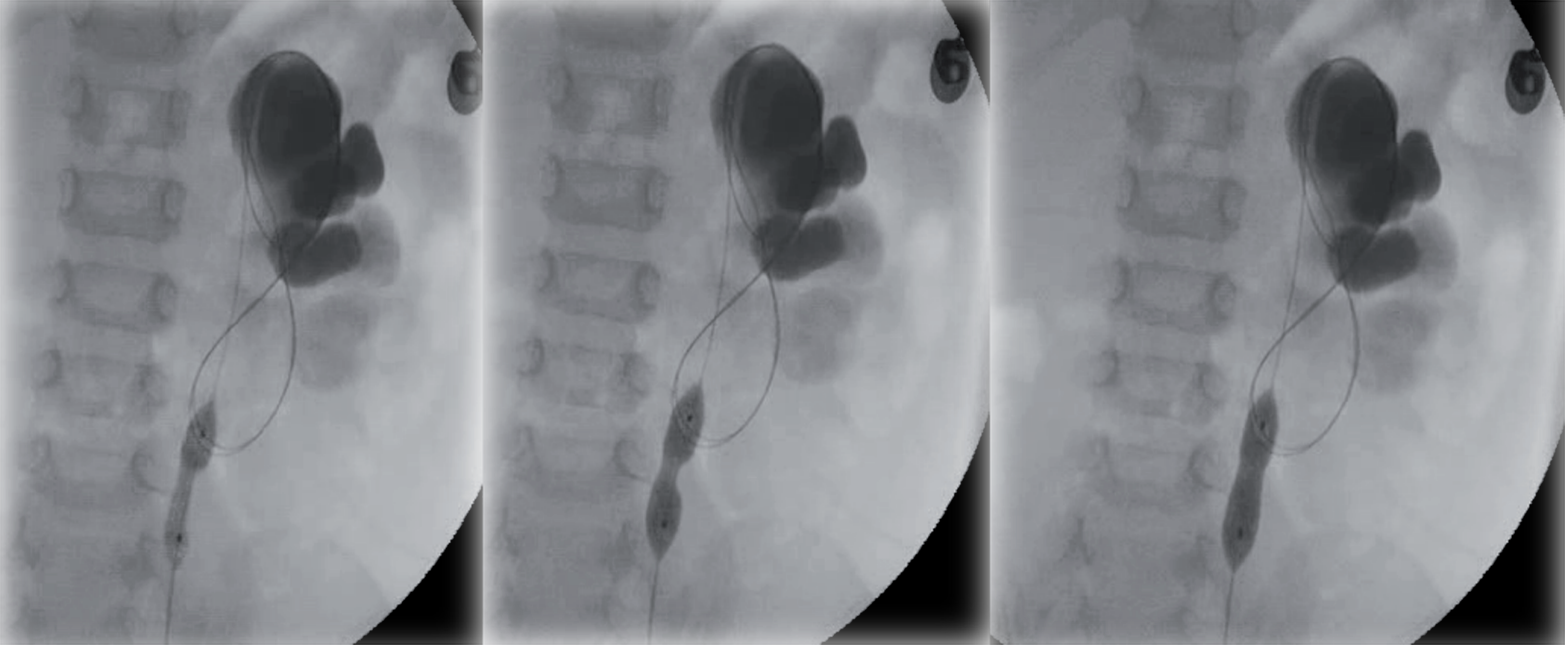
When no ‘waist’ is observed we considered this to represent extrinsic stenosis.

When no ‘waist’ is observed we considered this to represent extrinsic stenosis (Figure-2) and a laparoscopic VH was performed.

### “Vascular Hitch”

**Figure 3:**
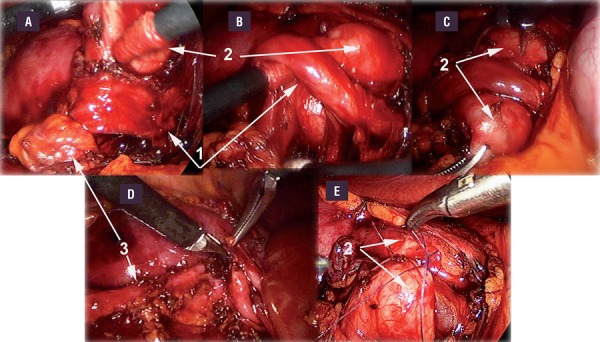
Cranial relocation of lower pole crossing vessels or "vascular-hitch". A-B: The lower pole vessels were dissected free from the PUJ; C-D: full mobility of the pelvis and PUJ was confirmed by the ‘shoe shine’ manoeuvre; E: The lower pole vessels were then fixed in a cephalic position away from the PUJ by suturing the pelvis on either side of the vessels with two to three absorbable sutures without tension. 1: Crossing vessels; 2: Renal pelvis; 3: Ureter.

This technique consists of a laparoscopic transperitoneal exposure of the lower pole vessels with the patient in a lateral position. Three 5mm ports were used in all cases. The lower pole vessels were dissected free from the PUJ and full mobility of the pelvis and PUJ was confirmed by the ‘shoe shine’ manoeuvre. The PUJ was carefully inspected for intrinsic stenosis, and inspection for evidence of peristalsis across the junction was performed. To remove doubt, the pelvis was also distended with saline from a fine-bore needle to assess drainage across the PUJ. The lower pole vessels were then fixed in a cephalic position away from the PUJ by suturing the pelvis on either side of the vessels with two to three absorbable sutures without tension (Figure-3).

## RESULTS

Six children (4m, 2f) were included in the study. Median age at presentation was 10.8 years (6-15 years) and the median duration of symptoms was 22 months (6 months-4 years). All children had intermittent loin pain, one presented with a UTI and one had haematuria. Grade IV hydronephrosis was demonstrated on USS in 4 cases (with mild parenchymal thinning) and grade III in 2. The anteroposterior diameter of the pelvis was 41mm (60-22mm). In all cases an obstructive renogram was found, 3 retaining preserved function and 2 with mild loss of function (33% and 37%).

The right kidney was affected in 3 patients and left kidney in 3. The median duration of surgery was 99 min (85-110 min) and mean hospital stay was 24h in all cases. In no case was a drain or double J stent used. No intraoperative or postoperative complications were encountered.

All children remained symptom free at a median follow-up of 14 months (6-24). Routine ultrasound showed a decreased degree of hydronephrosis and anteroposterior diameter of the pelvis in all cases with a median value of 14mm (7-20mm). MAG3-lasix renograms demonstrated improved drainage in all children and no change in renal function (Table-1).

**Table 1:** Patient data.

Age	Preoperative hydronephrosis grade	Preoperative APD renal pelvis	Preoperative parenchymal thinning	Preoperative differential renal function	Preoperative symptoms	Postoperative hydronephrosis grade	Postoperative APD renal pelvis	Postoperative differential renal function	Postoperative curve renogram	Duration of follow-up (months)
13	IV	60	+	45	Pain	II	20	47	Normal	20
11	IV	40	+	43	Pain	I	7	48	Normal	24
15	IV	47	++	36	Pain	II	13	40	Normal	11
9	III	22	-	45	Pain + UTI	II	11	47	Normal	9
6	IV	34	+	31	Pain + haematuria	III	20	35	Semiobstructive	6
12	IV	55	++	30	Pain	II	23	31	Normal	12

**APD =** Anteroposterior diameter

**UTI =** Urinary tract infection

## DISCUSSION

Although prenatal hydronephrosis and hydronephrosis diagnosed in childhood or adolescence may appear to be the same condition, doubts remain about whether it is actually the same entity. Thus, the need to differentiate the aberrant polar vessels associated with intrinsic UPJ stenosis or diagnosing a polar vessel as a single extrinsic cause of obstruction becomes relevant in the older age group.

PUJ obstruction diagnosed antenatally or in the first few years of life usually have an intrinsic stenosis as the cause of their obstruction. In these patients, the existence of a polar vessel is treated as an incidental finding (7, 8). Those that present in late childhood or adulthood generally report intermittent symptoms and a significant proportion are associated with accessory lower pole vessels (9, 10). The debate rests in whether these vessels are merely an anatomical variation with no pathological significance or whether they play a role in the pathogenesis of the outflow impairment or obstruction at the PUJ level.

Histological studies examining intrinsic obstruction have postulated that the obstruction is caused by aperistaltic segments with abnormal amounts of muscle and collagen deposition (11).

Extrinsic obstruction is thought to originate from an overlying renal vessel. Whether the vessel alone causes obstruction or whether there is also a component of underlying UPJ fibrosis remains unclear and controversial (12). Some studies have confirmed that muscle density significantly increases in those with CV obstruction compared to intrinsic obstruction (13, 14). This may mean that extrinsic stenosis produces different histological changes in the UPJ when compared to intrinsic stenosis. However, it is not clear if these changes later form obstructions.

Numerous imaging modalities have been used to identify crossing renal vessels, such as angiography, endoluminal ultrasound, Doppler ultrasound, spiral CT, and MRI (1). However, none can prove whether crossing renal vessels are obstructive or are merely incidental findings (15). Many paediatric urologists believe that during a dismembered pyeloplasty in older symptomatic children with lower pole vessels, there is a lack of a macroscopically obvious intrinsic obstruction at the PUJ (3).

Some research groups perform a VH if the patient meets certain criteria: a normal calibre ureter, a normal appearance UPJ without stenosis or narrowing and good peristalsis across the UPJ (4). Other groups perform a water overload test intraoperatively after freeing the lower pole vessels. This consists of inducing hydronephrosis using intravenous hyperhydration and diuretic medications injected during the first stages of the procedure (5).

We hypothesise that the calibration of the PUJ with a balloon under radiological screening while assessing the presence of intrinsic stenosis is a reliable and safe way to decide which patients may benefit from the VH.

Although it may be felt that calibration may prolong the procedure, this was not demonstrated in our series. Our operative time is comparable to other series recently published (3-5). There is no increase in complication rates or postoperative hospital stay associated with this modification. While these steps may increase the cost of the procedure the costs of operation for stent removal are avoided. Therefore, we think that assessment of the PUJ with a dilating balloon intraoperatively is a useful tool to avoid inappropriate surgical technique being employed.

From our previous experience dilating the UPJ endoscopically in infants with PUJO we believe that intrinsic stenosis always demonstrates an indentation or ‘waist’ visible in the high-pressure balloon under radiological screening. This narrowing requires a pressure of over 8-10atm (16) in the balloon to overcome it. In patients with extrinsic stenosis this balloon inflation did not show any indentation or waist.

Previous descriptions of “vascular hitch” have used exclusively laparoscopic observation of the ureteropelvic junction to decide there is no intrinsic cause of stenosis and therefore perform a VH. In our series, in addition to internal or endourologic observation we also examine the ureteropelvic junction radiologically (external observation), using additional information to decide on when purely extrinsic stenosis is present (4, 5). We believe that this assessment of the PUJ increases the safety and accuracy of the surgical decision making process.

Despite the small number of patients in this study, the postoperative results are satisfactory after a follow-up period longer than 6 months in all cases. A large number of patients is now required to provide further reliability to the calibration process for the diagnosis of intrinsic stenosis.

We feel that the additional steps described here do not add any increased morbidity to this operation, instead we believe they benefit the patient by ensuring that only the procedures required are performed. Retrograde instrumentation of urinary tract prior to laparoscopic pyeloplasty is still used by some groups to insert a double J (17), this has been reported as adding technical difficulty to pyeloplasty suture. Our approach is safe, as it has not increased the difficulty of the procedure and no intraoperative or postoperative complications were registered. Our success in calibrating the PUJ relies on consistent technique and adequate instrumentation.

The limitations of this study are that it is a retrospective study without a control group. The absence of the control group (laparoscopic “vascular hitch” in the presence of an existrinsic “waist” during calibration) is due to our feeling that to offer VH without assessing for intrinsic obstruction leaves open the potential for repeat surgery to be required at a later date that could have been avoided had this balloon assessment being completed. The small number of patients make this study statistically underpowered and represents a challenge to validate the technique.

## CONCLUSION

Assessment of PUJ with high-pressure balloon calibration in patients with PUJO and crossing vessels may allow us to better differentiate between those cases with extrinsic stenosis of the PUJ from those with associated intrinsic obstruction. Consequently, these patients would receive a less invasive surgery preserving the integrity of the urinary tract and avoiding the need for stenting, reducing their associated morbidity.

In these patients, laparoscopic transposition of lower pole crossing vessels (‘vascular hitch’) may be a safe and reliable surgical technique.
